# Comparing total hip arthroplasty and hemiarthroplasty for the treatment of displaced femoral neck fracture in the active elderly over 75 years old: a systematic review and meta-analysis of randomized control trials

**DOI:** 10.1186/s13018-020-01725-3

**Published:** 2020-06-11

**Authors:** Yijun Liu, Xiaokun Chen, Peixun Zhang, Baoguo Jiang

**Affiliations:** Department of Orthopaedics and Trauma, Peking University People’s Hospital, No. 11 Xizhimen South Street, Xicheng District, Beijing, 100044 China

**Keywords:** Displaced femoral neck fractures, Total hip arthroplasty, Hemiarthroplasty

## Abstract

**Background:**

Displaced femoral neck fractures (DFNF) are increasingly common in elderly patients. Hip arthroplasty, the recommended treatment of DFNF, consists of the total hip arthroplasty (THA) and hemiarthroplasty (HA). THA is superior to HA in younger patients. However, there are concerns whether the more substantial surgical trauma and higher dislocation rate would trade off the advantages of THA due to frailty and lower physical demands in the elderly over 75 years.

**Methods:**

We conducted the literature search by searching PubMed, Embase, the Cochrane Library, ClinicalTrials.gov, and Web of Science from the inception dates to June 1, 2019. Randomized controlled trials (RCTs) were included according to the inclusion and exclusion criteria. Included studies were analyzed according to Cochrane review methods.

**Results:**

Nine studies met the inclusion criteria totaling 631 participants (301 THA and 330 HA). Four of the studies conducted were identical to a previous study but look at different follow-up periods. Our study revealed that THA was superior in terms of pain HHS, total HHS, EQ-5D, and acetabulum erosion, with a trend of a lower mortality rate within 6 months after surgery. However, the THA group had a longer average operative time and higher dislocation rate, with a trend towards a higher general complication rate. Moreover, there was no significant difference in terms of reoperation rate, postoperative infection, peri-prosthetic fracture, and VTE prevalence across the groups.

**Conclusions:**

THA may be a preferred management option for active elderly patients over 75 years old, which can provide superior hip function and life quality with acceptable risks. Strict management should be followed to prevent dislocation following a THA, especially within the first 6 months.

**Trial registration:**

This study was registered at the International Prospective Register of Systematic Reviews (CRD42019139135).

## Background

Hip fractures are a worldwide health problem, with the incidence increasing over time. The prevalence of hip fractures in Asian countries will rise from over 1 million per year in 1990 to over 6 million by 2050 [1]. The residual lifetime risk of suffering a hip fracture is about 5.6% for men and 20% for women [2]. The femoral neck fracture (FNF) is a major type of hip fracture, whose treatment includes internal fixation, HA, or THA [3]. Internal fixation is a preferred management option for young people or the elderly who are intolerant of prosthesis surgery [4]. THA and HA are widely used in DFNF elderly [3]. In general, HA has advantages of the shorter operation time, less blood loss, less technical demand, less economic burden, and a lower dislocation rate [5–9]. THA is associated with better hip function, less acetabulum erosion, and a lower revision rate [5–10].

With the increase of longevity and activity level, today’s elderly have a higher demand for adequate hip function and a higher risk of acetabulum erosion after arthroplasty than before, which seems to favor the THA procedure [11–13]. Some RCTs [12–16] suggested that THA could provide a superior hip function without increasing the mortality rate and general complication rate in patients with a mean age over 75 years old. Current guidelines [17, 18] suggested that THA would provide greater benefit in selected elderly patients because of less pain and lower revision rate, such as patients who were able to walk independently, not cognitively impair, and medically fit for the procedure.

However, the frequency of HA procedures increases with age, whose cutoff is at 76, due to, in part, the contradictions between the more extensive surgery of THA and the lower surgical tolerance of elderly patients [19, 20]. Some authors [6, 21–23] found that there was no significant difference in hip function and life quality between the two procedures in patients with a mean age over 75 years, with better prosthetic survivorship and a trend of fewer hip complications in the HA group. A systematic review [8] indicated that both procedures are reasonable in patients over 80 years because of the nonsignificant differences in hip function, reoperation rate, and mortality rate.

There are still concerns whether the more substantial surgical trauma and higher dislocation rate would trade off the advantages of THA due to frailty and lower physical demands in the elderly over 75 years [6, 8, 19, 21]. Therefore, the choice of THA or HA in DFNF in this population remains uncertain. The previous meta-analyses did not include strict age restrictions, and some of these studies also included patients who were unable to walk independently or received a polycarbonate–urethane (PCU) THA that would lead to high heterogeneity, therefore making it complicated to apply these findings to patients with older age [7, 8, 24–27]. The purpose of this study is to compare the effectiveness and safety of THA and HA in the active elderly over 75 years by using the latest evidence from previously performed RCTs.

## Methods

This review was performed according to the Systematic Reviews and Meta-Analyses (PRISMA) statement [28]. This study was registered at the International Prospective Register of Systematic Reviews (CRD42019139135). The date of registration is 16 June 2019.

### Search strategy

We conducted the literature search by searching PubMed, Embase, the Cochrane Library, ClinicalTrials.gov, and Web of Science from the inception dates to June 1, 2019 (Additional file 1). Other search methods included a manual search using the references in relevant articles and abstracts of related meetings, and personal communication with experts in the field. We applied no language restrictions. We used the following combined texts and MeSH terms: femoral neck fractures, hemiarthroplasty, total hip arthroplasty, RCT, and entry terms.

### Inclusion and exclusion criteria

RCTs were regarded as eligible if they met the following inclusion criteria: (1) trials enrolling elderly patients and mean age > 75 years old, (2) trials enrolling community-ambulant patients, (3) trials enrolling DFNF (Garden III, IV), and (4) trials comparing THA and HA. Exclusion criteria were as follows: (1) bedridden or immobile patients, (2) patients with advanced radiological osteoarthritis or rheumatoid arthritis in the fractured hip, and (3) patients with pathological fractures secondary to malignant disease.

### Research quality assessment

Two investigators (YJL, XKC) assessed the bias risk of each included study according to the Cochrane risk-of-bias criteria independently [28, 29]. We defined the other bias as not enough detailed description of materials, methods, and results; not similar in baseline characteristics; and sponsored by companies.

### Data extraction

Two independent investigators (YJL, XKC) reviewed the titles and abstracts of the search results for trial selection. If there was insufficient information to determine if the study should be selected, investigators read the full text for further assessment. Data extraction was also performed by these two investigators (YJL, XKC). Any disagreements were resolved by a third independent investigator (PXZ). We extracted the following information from each study: lead author, participant characteristics, follow-up duration, details of the interventions, the primary outcomes, and secondary outcomes. The primary outcomes measured were intra-operative details (blood loss and duration of operation), mortality, functional outcomes (Harris Hip Score), reoperation rates, dislocation rates, erosion rates, infection rates, deep venous thrombosis prevalence, and general complication rates. The secondary outcomes were prosthesis loosening, peri-prosthetic fractures, Europol (EQ) index-5D score, and the duration of hospital stay.

### Statistical analysis

We used both the Q2 test and *I*^2^ test to assess the statistical heterogeneity among studies. If the *P* value resulting from the Q2 test was < 0.1 or the value of *I*^2^ test was > 50%, it was indicative of statistical heterogeneity. We calculated the relative risks (RR) for dichotomous outcomes and weight mean difference (WMD) or standardized mean difference for continuous outcomes with corresponding 95% confidence intervals (CI). The results were pooled by a fixed effects model. If data was indicative of statistical heterogeneity, a random effects model was applied instead. If two studies were based on the same population, only the latter one was included in the forest plot. We performed the sensitivity analysis by excluding research to evaluate the stability of the results. Subgroup analysis would be conducted to evaluate heterogeneity. Narrative synthesis would be used by summarizing characteristics on the table when quantitative synthesis was not available. If more than ten studies were included, funnel plots would be used to assess publication bias. The quality of key outcomes would be assessed by Grading of Recommendations Assessment (GRADE) [30]. We used the Cochrane Review Manager 5.3 software for data analysis, and *P* < 0.05 was statistically significant.

## Results

### Study characteristics

**Fig. 1:**
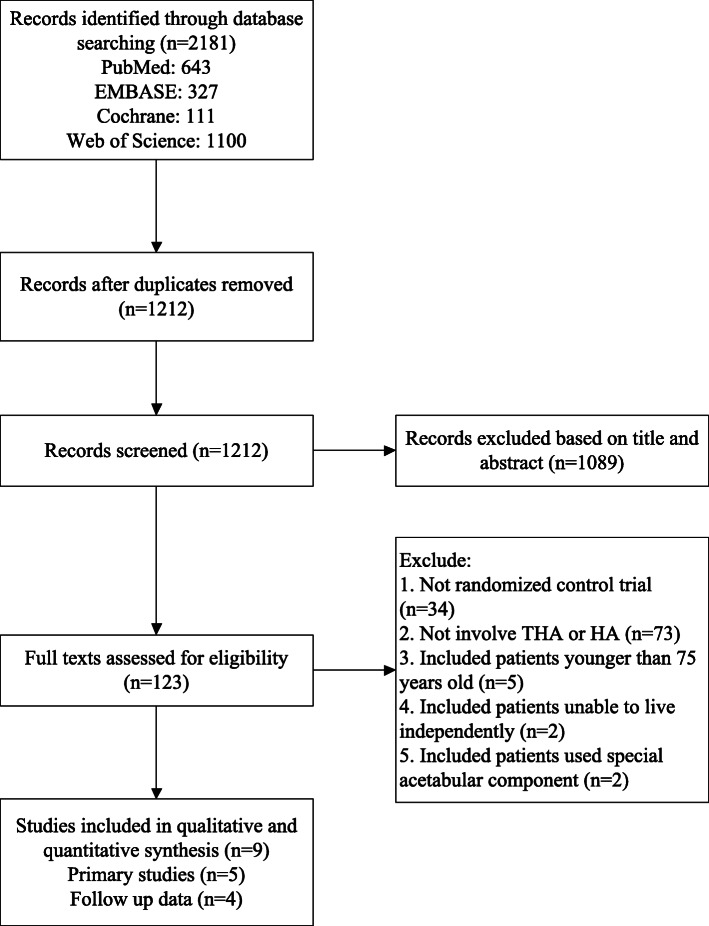
PRISMA flowchart of study selection

We identified 2186 studies, of which nine studies met the inclusion criteria [6, 9, 12–16, 21, 22]. Four of them were the same as one of the studies, but with different follow-up [9, 12, 16, 21] (Fig. 1). During study selection, we excluded some RCTs, including five studies that incorporated patients younger than 75 years old [5, 31–34], and two studies included patients unable to live independently [35, 36], and two studies performed the THA procedure with PCU acetabulum [37, 38]. There were a total of 631 participants in the nine trials included in the study. Of these, 301 patients underwent the THA procedure, and 330 patients underwent the HA procedure. All trials were published between 2006 and 2017 (Table 1). The range of the duration of follow-up was from 24.0 to 194.0 months. One of these studies also included a third arm (internal fixation), but the data from these arms were not taken into account [14].

**Table 1:** Characteristics of the included studies

Author	Year	Location	No., THA/HA (P)	Garden classification	Mean age, THA/HA (years)	THA details	HA details	Operative time, THA/HA (min)	Mean follow-up duration (months)	Reoperation causes	Risk factors of dislocation
Keating	2006	Scotland	69/69	TypeIII/IV	75.2/75.0	Cemented femoral component	Bipolar, cemented	NA	24	THA: 3 for dislocation, 2 for infection, 1 for wound dehiscence. HA: NA	Posterior exposure
Avery and Baker	2011 and 2006	UK	40/41	TypeIII/IV	74.2/75.8	Cemented acetabular and femoral component	Cemented	NA	108	THA: 1 for femoral stem subsidence. HA: 3 for acetabular erosion, 1 for peri-prosthetic fracture	NA
Hedbeck and R. Blomfeldt	2011 and 2007	Sweden	60/60	TypeIII/IV	80.5/80.7	Cemented acetabular and femoral component	Bipolar, cemented	120/78	48.4	THA: 2 for infection. HA: 1 for peri-prosthetic fracture	Severe cognitive dysfunction
Macaulay and Macaulay	2008 and 2008	The USA	17/23	TypeIII/IV	82/77	NA	Unipolar or bipolar, and cemented or uncemented	89/82	34	NA	NA
Tol and van den Bekerom	2017 and 2010	Netherlands	115/137	TypeIII/IV	82.1/80.3	Cemented acetabular and femoral component	Bipolar, cemented	NA	144	THA and HA: 5 for loosening of the prosthesis, 2 for acetabular erosion, 1 for deep infection	Posterolateral approach (compared with anterolateral approach)

*NA* not available

### Risk of bias assessment

**Fig. 2:**
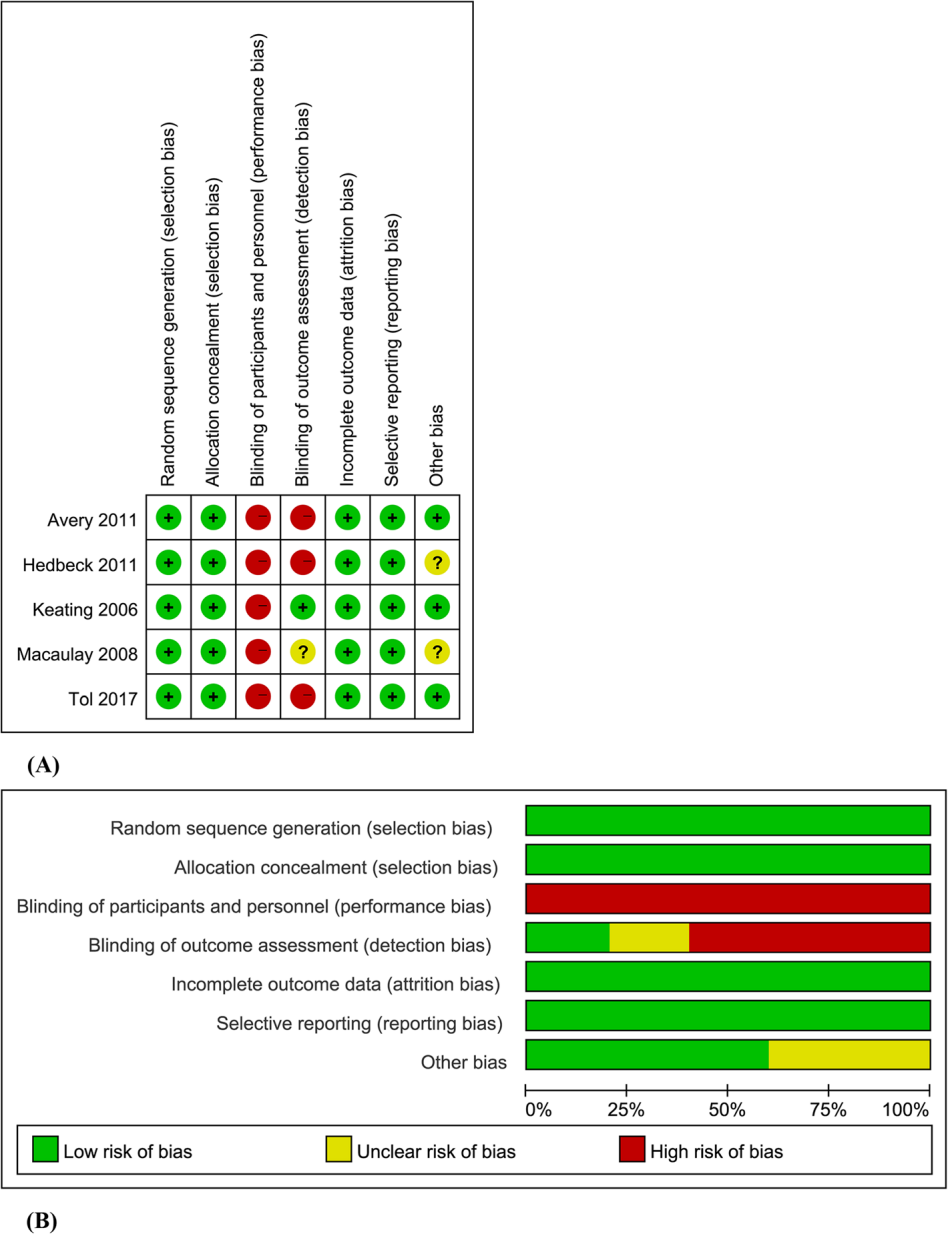
Risk of bias graph (**a**) and summary (**b**) of included studies

All nine randomized controlled trials were assessed and found to have used adequate randomization procedures, including the sealed-envelope technique, computer randomization programs, and systemic random sampling [6, 9, 12–16, 21, 22]. Only one of the studies utilized blinding of outcome measurements, which had the interviewers blinded to the patient recruitment and randomization or the type of surgery performed [14]. There was no blinding of participants in any of the included studies. Four of the studies were multi-center studies [13, 14, 16, 21]. One of the studies was funded by a commercial entity [22]. The results of the risk of bias assessment were summarized in Fig. 2. The quality assessment of key outcomes was shown in Additional file 2.

### Operative time

**Fig. 3:**
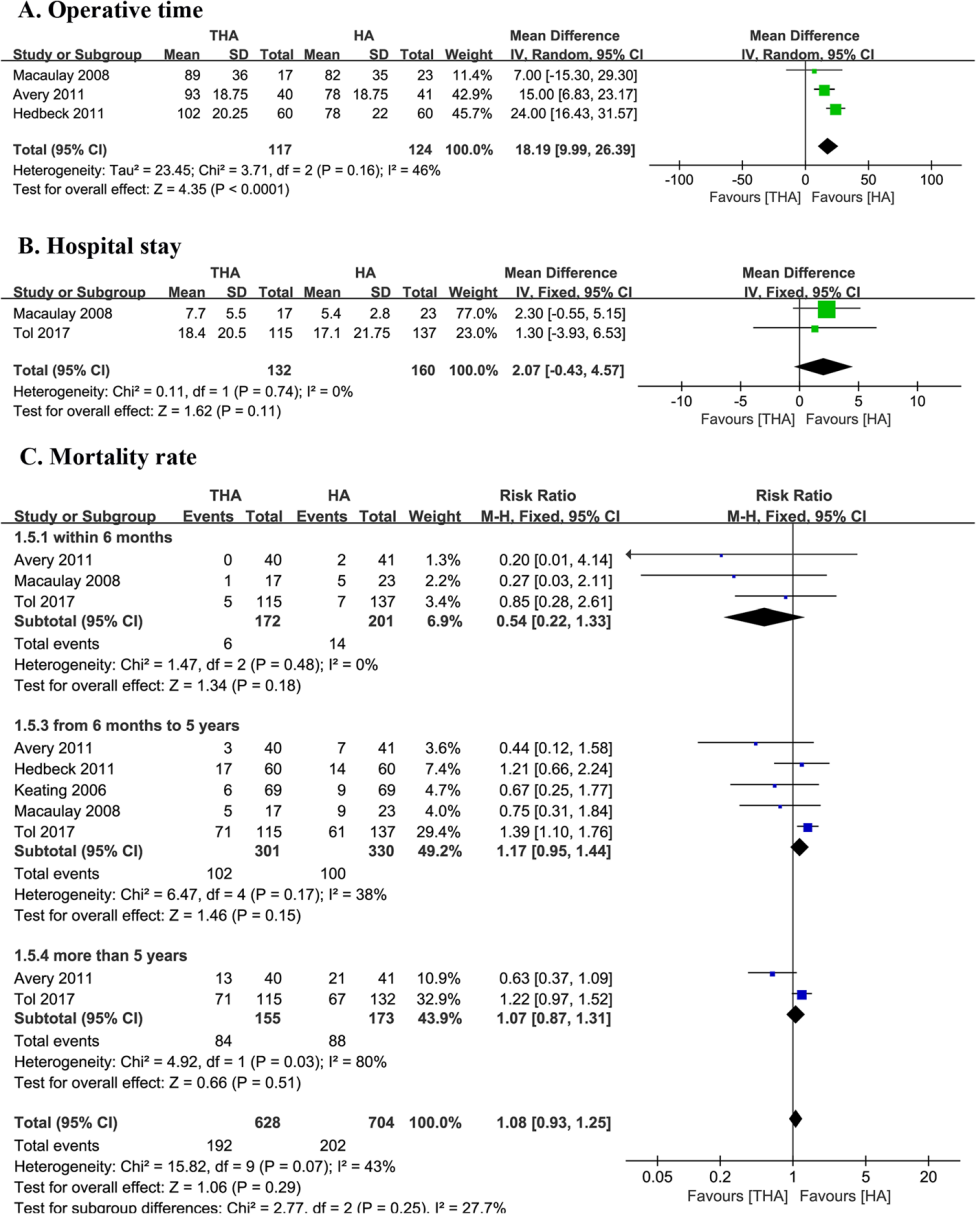
Forest plot of comparison of operative time, hospital stay, and mortality rate

Three studies assessed the operative time in both the THA and the HA groups (117 with THA and 123 with HA) [13, 16, 22]. The operative time was significantly longer in the THA group (MD 18.20, 95% CI 9.99–26.41, Fig. 3), and heterogeneity across the studies was 46%.

### Mortality rate

**Fig. 4:**
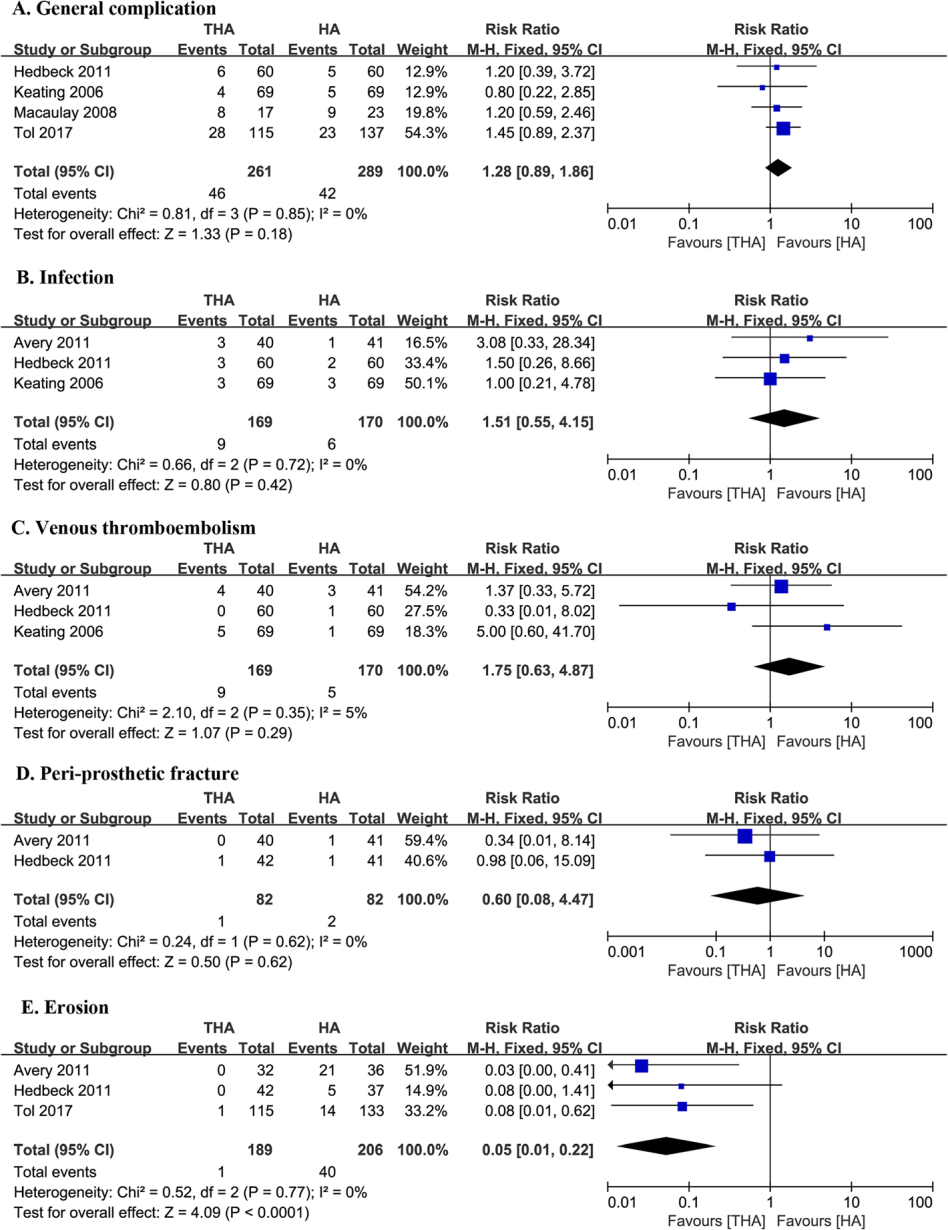
Forest plot of comparison of complications

Five studies assessed the mortality rates of both groups (301 with THA and 330 with HA) [13, 14, 16, 21, 22]. We divided the data into three subgroups based on the follow-up duration (within 6 months, between 6 months and 5 years, and more than 5 years). Three studies assessed the mortality rates within 6 months of follow-up (*I*^2^ = 0%) [13, 16, 21]. The mortality rate was 5.7% in the THA group and 9.2% in the HA group. Although there was a lower mortality rate in the THA group, it was found that there was no significant statistical difference between the two groups (RR 0.54, 95% CI 0.22–1.33, Fig. 3). There was also no significant statistical difference in the 6 months to 5 years group (RR 1.17, 95% CI 0.95–1.44, Fig. 4) [13, 14, 16, 21, 22] and the more than 5 years follow-up group (RR 1.07, 95% CI 0.93–1.24, Fig. 3) [13, 21].

### General complication

Four studies assessed the general complication rates in both groups (261 with THA and 289 with HA), with the mean follow-up being 14.5 months [14, 16, 21, 22]. Although the HA group had lower rates of general complications (*I*^2^ = 0%), there was no significant difference from the THA group (RR 1.28, 95% CI 0.89–1.86, Fig. 4).

### Infection rate

Three studies assessed the infection rates of both groups from 339 interventions (*I*^2^ = 0%) [13, 14, 22]. Pooling the data of the included studies elicited no significant statistical difference between the THA and HA groups (RR 1.51, 95% CI 0.55–4.15, Fig. 4).

### VTE

Three studies assessed the venous thromboembolism (VTE) prevalence in both groups from 339 interventions (*I*^2^ = 5%) [13, 14, 22]. No significant difference was found between the groups (RR 1.75, 95% CI 0.63–4.87, Fig. 4).

### Peri-prosthetic fracture

Two studies assessed the rate of peri-prosthetic fracture in both groups from 164 interventions (*I*^2^ = 0%) [13, 22]. There was no significant statistical difference between both groups (RR 0.6, 95% CI 0.08–4.47, Fig. 4).

### Erosion

Three studies reported the erosion rates in both groups (189 with THA and 202 with HA). The mean duration of follow-up was 45.3 months [13, 21, 22]. Erosion rates were 0.48% in the THA group and 13.7% in the HA group with a significant difference (RR 0.05, 95% CI 0.01–0.20, Fig. 4) and no significant between-study heterogeneity (*I*^2^ = 0%).

### Reoperation rate

Four studies assessed the relative risk of reoperation rate in both two groups (284 with THA vs. 307 with HA) [13, 14, 21, 22]. We divided the data into two subgroups according to follow-up durations (within 5 years and more than 5 years). Four studies assessed the reoperation rate within 5 years of follow-up (*I*^2^ = 0%) [13, 14, 21, 22]. Pooling the data revealed no significant difference (RR 0.94, 95% CI 0.43–2.04) across the groups. Two studies assessed the reoperation rate after 5 years of follow-up (*I*^2^ = 0%) [13, 21]. The reoperation rate was 2.6% in the THA group and 5.6% in the HA group; however, there was no significant difference (RR 0.45, 95% CI 0.14–1.39) between the two procedures. If we added the patients with painful symptoms to the group of patients revised, the reoperation rate was significantly lower in the THA group after 5 years of follow-up (RR 0.29, 95% CI 0.1–0.85) without heterogeneity (*I*^2^ = 0%) [13, 21]. The causes of reoperation are summarized in Table 1.

### Dislocation rate

**Fig. 5:**
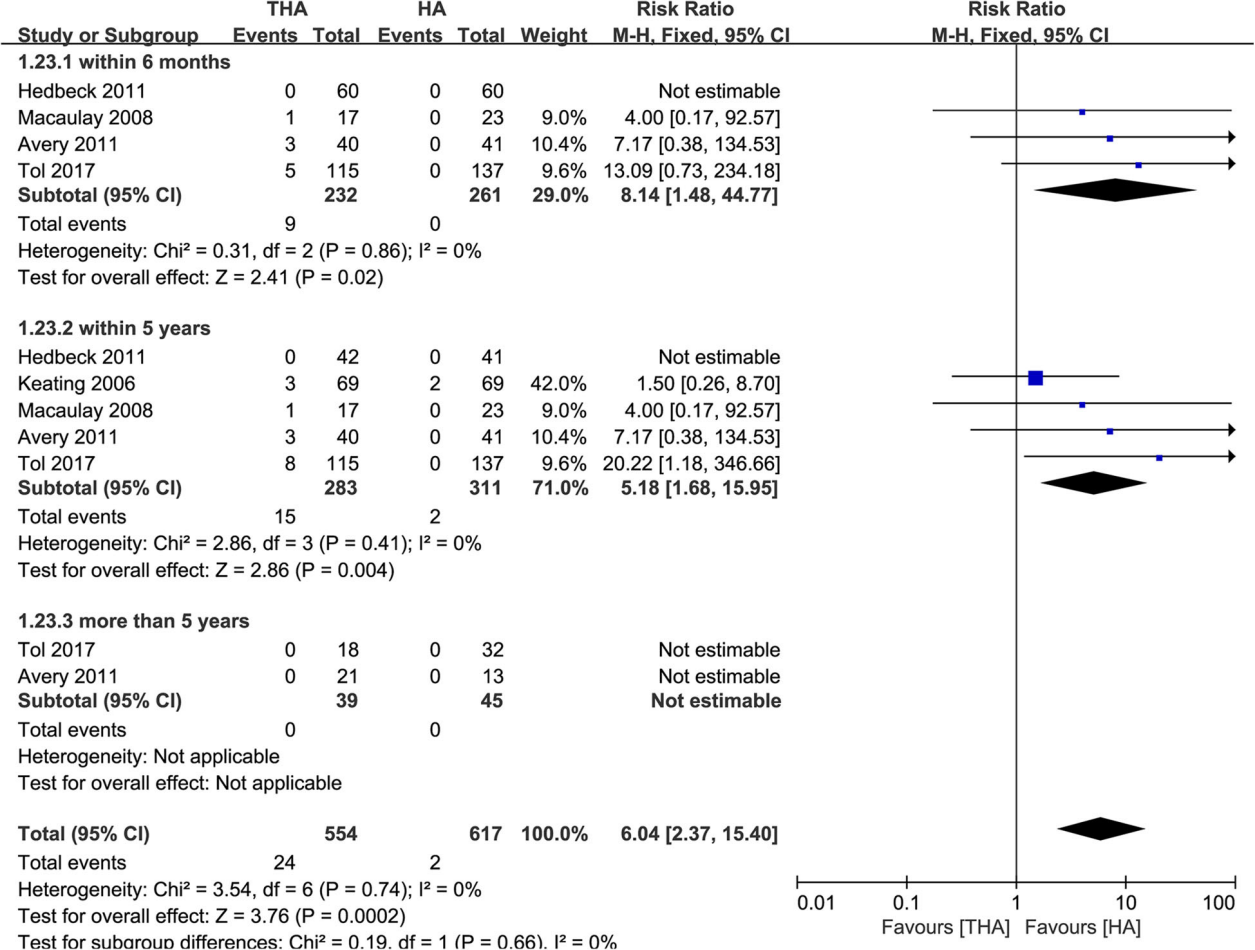
Forest plot of comparison of dislocation

Five studies reported the dislocation rate in both groups (301 with THA and 330 with HA). We divided the data into three subgroups according to follow-up duration (within 6 months, within 5 years, and more than 5 years) [13, 14, 16, 21, 22]. There was a significant statistical difference between dislocation rates in the THA group and the HA group within the first 6 months (RR 8.14, 95% CI 1.48–44.77, Fig. 5) and within 5 years of follow-up (RR 5.18, 95% CI 1.68–15.95). The heterogeneity was 0% in both subgroup analyses. There were no dislocations from 84 interventions reported 5 years following the surgery [13, 21]. The risk factors of dislocation are summarized in Table 1.

### Total HHS

**Fig. 6:**
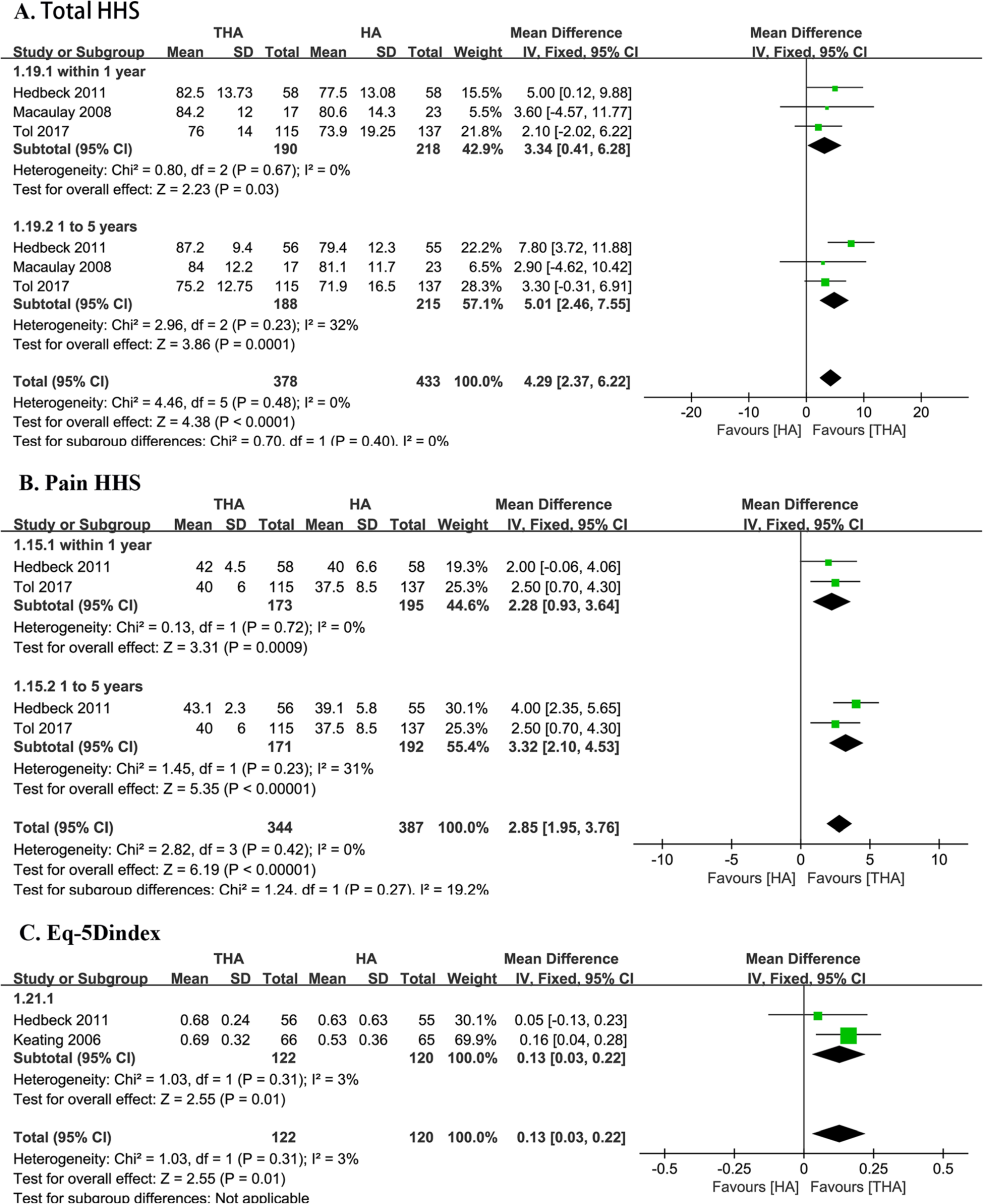
Forest plot of comparison of hip function and life quality

Three studies assessed the total Harris Hip Score (HHS) in both groups (190 with THA and 218 with HA) [16, 21, 22]. We divided the data into two subgroups according to the follow-up duration (within 1 year and from 1 to 5 years). Three studies assessed the total HHS within 1 year (*I*^2^ = 0%) [16, 22]. Pooling the data showed a significantly higher total HHS in the THA group (MD 4.63, 95% CI 0.44–8.82, Fig. 6). Two studies assessed the total HHS from 1 to 5 years after surgery (*I*^2^ = 0%), and the data favored the THA group (MD 4.91, 95% CI 2.73–7.08) [16, 22].

### Pain HHS

Two studies assessed the HHS pain in both groups (173 with THA and 195 with HA) [21, 22]. We divided the data into two subgroups according to the follow-up duration (within 1 year and from 1 to 5 years). Two studies assessed the HHS pain within 1 year (*I*^2^ = 0%) [21, 22]. Patients in the THA group experienced significantly less pain (MD 2.28, 95% CI 0.93–3.64, Fig. 6). Two studies assessed the HHS pain from 1 to 5 years of follow-up (*I*^2^ = 31%), and the data favored the THA group (MD 3.32, 95% CI 2.10–4.53, Fig. 6 )[21, 22].

### Function HSS

Two studies assessed the HHS function in both groups (173 with THA and 192 with HA) [21, 22]. We divided the data into two subgroups according to the follow-up duration (within 1 year and from 1 to 5 years). Two studies assessed the HHS function within 1 year (*I*^2^ = 57%), and the data tended to favor the THA group (MD 1.39, 95% CI − 1.52–4.30) [21, 22]. Two studies assessed the HHS function from 1 to 5 years of follow-up (*I*^2^ = 35%), and the data tended to favor the THA group (MD 2.79, 95% CI − 0.04–5.61) [21, 22].

### EQ-5D_index_

Two studies assessed the EQ-5D in both groups with a mean follow-up duration of 18 months (122 with THA and 120 with HA) [14, 22]. The THA group had better EQ-5D_index_ score (MD 0.13, 95% CI 0.03–0.22, Fig. 6). There was no significant heterogeneity (*I*^2^ = 3%).

### Hospital stay

Two studies assessed the length of hospital stay in both groups from 292 interventions (*I*^2^ = 0%). The data tended to favor the HA group (MD 1.30, 95% CI − 0.43–4.57, Fig. 3) [16, 21].

## Discussion

The choice of THA or HA in DFNF in the active very elderly (over 75 years) remains uncertain [7, 8, 13, 14, 16, 24–27]. Our study revealed that THA was superior in terms of acetabulum erosion, pain HHS, total HHS, and EQ-5D, with a trend of a lower mortality rate within 6 months after surgery. However, the THA group had a longer average operative time and a higher dislocation rate, with a trend towards higher general complication rates, and longer duration of hospital stays. Moreover, there was no significant difference in terms of reoperation rate, postoperative infection, peri-prosthetic fractures, and VTE prevalence across the groups.

Compared to other meta-analyses [7, 8, 24–27], our study limited patients’ age to over 75 years old, which was more specific than the previous studies. We excluded two RCTs due to the inclusion of patients with compromised mental states [35, 36], and two RCTs because they used a THA procedure utilizing the PCU acetabulum, which was pliable, and therefore might increase the erosion rates and negatively affect stability. This method also led to differences in operative time, hip function, and complication rates between PCU-THA and traditional THA [37, 38]. To our knowledge, this is the first meta-analysis comparing THA and HA in the active elderly over 75 years. The strict inclusion criteria reduced heterogeneity significantly in essential outcomes, such as pain and total HHS when compared to other meta-analyses [7, 8, 24].

We found no significant difference in the reoperation rate between two groups, which was supported by all the RCTs included [6, 9, 12–16, 21, 22]. However, meta-analyses without strict inclusion criteria on age, THA component, or mental states suggested that reoperation rates were higher in the HA groups [7, 8, 24, 26, 27, 39]. Kannan et al. [23] found THA with an increased reoperation rate compared to HA according to the national registries, which would be lower with increasing age. The negative finding of our study may result from less physical demands and lower surgical tolerance in this population. After adding in the patients with painful symptoms, the reoperation rate was significantly lower in the THA group after 5 years follow-up, which had the following implications. First, the acetabular wear of HA would lead to painful symptoms despite the lower physical demand. Second, patient frailty may make it less likely to perform reoperation. Third, patients over 75 years in the THA group might have a better quality of life in the long term [35].

Although all the RCTs included except van den Bekerom et al. [6] found no significant difference in dislocation rate between two procedures in patients over 75 years [9, 12–16, 21, 22], we found a higher dislocation rate in the THA group (5.3% vs. 0.64%), which was supported by other studies [6–8]. A recent systematic review [8] suggested that the dislocation rate in patients over 50 years was 8.1% and 2.7% in the THA and HA group, respectively. We speculate that the lower trend of dislocation rate in patients over 75 years is mainly due to the lower level of activity. We believe the dislocation rate of THA, which is 3.9% within 6 months and 5.3% within 5 years, is acceptably low [14]. 82.4% of the patients with dislocation could be treated conservatively [13, 14, 16, 21, 22]. Moreover, our results suggested that the dislocation risk of THA was higher within 6 months after surgery. Avery et al. [13] found that all dislocations in the THA group occurred within 1 month after surgery. van den Bekerom et al. [6] reported that 62.5% of the dislocations occurred in the hospital-stay duration. Additionally, we found no dislocation occurs in the 84 patients with a follow-up of more than 5 years (mean 129 m), which was not mentioned in other meta-analyses [7, 8, 25–27]. We theorize that first, dislocation may be a risk factor for mortality in patients over 75 years [21]. Second, patients with long-term follow-up have significantly lower levels of activity, which may contribute to the absence of dislocation.

In our series, the THA group had better total HHS outcomes in the short (1 year) and medium term (5 years). These results were supported by previous studies [12, 14–16, 22, 24, 25, 39]. Lewis et al. [8] found no significant difference in total HHS and pain HHS in patients over 80 years. However, they included some RCTs using PCU-THA, which might lead to high heterogeneity [37, 38]. Whether the superior hip function of THA in this population still exists in the long term is controversial, whereas there is not enough data for our study. Avery et al. [13] and Tol et al. [21] found no significant difference in HHS between the two groups at a mean follow-up of 9 and 12 years. Our trial found that the THA group had better pain HHS after both in the short term and the middle term, with low heterogeneity. The heterogeneity might be associated with the differences in surgical techniques and rehabilitation programs because the two RCTs had similar inclusion criteria, patients’ characteristics, and prosthesis types [21, 22]. These results were different from the previous meta-analyses with younger patients [7, 8]. Besides, we found no difference in the functional HHS across the groups. Previous RCTs also reported the same result in 1, 5, and 12 years of follow-up [6, 21]. Therefore, we theorize that although the lower physical demand of patients over 75 years, THA can still provide less pain even in the short term due to the fewer acetabular wear, which may mainly contribute to the superior in total HHS and the quality of life [24]. The EQ-5D_index_ score was higher in the THA group with a mean follow-up of 18 months, indicating that the improvement of hip function by THA could still significantly improve the quality of life despite the lower activity level [14].

This study had some limitations. First, we included a small number of studies because of the strict inclusion criteria, and as a result, some essential data endpoints (average blood loss and long-term HHS) could not be analyzed. Second, nearly all the included studies [13, 16, 21, 22] had defects in the procedure for the blinding of participants and outcome assessment, and one trial was funded by the insurance company [22], which may have skewed the results. Third, we could only perform subgroup analyses according to age because of not enough data for the subgroup analyses based on comorbidities and ASA score. Fourth, the included studies were published between 2006 and 2017, and during this period, there were many advancements in orthopedics, such as prosthetic equipment and hip function evaluation methods. Further research with an older population or subgroup analyses based on other factors reflecting patient frailty, such as comorbidities and ASA score, is needed.

## Conclusions

These findings suggest that THA may be a preferred management option for active elderly patients over the age of 75 years. First, the more extensive surgery of THA will not lead to detectable increases in mortality rate and general complications. Second, older patients, who have lower physical demand, can still benefit from THA in terms of hip function and quality of life. The major concern of THA is the higher risk of dislocation, especially in the first 6 months, which is acceptably low in this population.

## Supplementary information

**Additional file 1.** Searching in Embase (1974 to June 1st, 2019). We drafted a search strategy to be used for the Embase database as an example.

**Additional file 2.** Summary of the quality assessments of key outcomes based on the GRADE approach. We summarised the key comparisons in tables according to the Grading of Recommendations Assessment (GRADE).

## Data Availability

The datasets and materials are available from corresponding authors on reasonable request.

## Abbreviations

FNF: Femoral neck fracture
DFNF: Displaced femoral neck fractures
NICE: National Institute for Health and Clinical Excellence
HHS: Harris Hip Score
EQ-5D: Europol index-5D
RR: Relative risks
CI: Confidence intervals
VTE: Venous thromboembolism
PCU: Polycarbonate–urethane
