# Supplementary material for: Comparing total hip arthroplasty and hemiarthroplasty for the treatment of displaced femoral neck fracture in the active elderly over 75 years old: a systematic review and meta-analysis of randomized control trials
Source: J Orthop Surg Res. 2020 Jun 11;15:215. doi: 10.1186/s13018-020-01725-3 (PMC7291510; doi:10.1186/s13018-020-01725-3)
Supplement: Supplementary file 1 — Additional file 1. Searching in Embase (1974 to June 1st, 2019). We drafted a search strategy to be used for the Embase database as an example. [file 13018_2020_1725_MOESM1_ESM.docx]

**Additional file 1: searching in Embase (1974 to June 1^st^, 2019)**

--------------------------------------------------------------------------------

#1 ‘femoral neck fracture’ /exp 11562

#2 ‘femoral neck fracture’ ab, ti 3125

#3 ‘femoral neck fractures’ ab, ti 3400

#4 ‘femur neck fracture’ ab, ti 100

#5 ‘femur neck fractures’ ab, ti 114

#6 #1 OR #2 OR #3 OR #4 OR #5 12392

#7 ‘hip hemiarthroplasty’/exp 643

#8 ‘hemiarthroplasties’ ab, ti 431

#9 ‘hemi-arthroplasty’ ab, ti 174

#10 ‘hemi arthroplasty’ ab, ti 174

#11 ‘hemi-arthroplasties’ ab, ti 28

#12 ‘hemiarthroplasty’ ab, ti 3127

#13 #7 OR #8 OR #9 OR #10 OR #11 OR #12 3659

#14 ‘total hip replacement’/exp 1041

#15 ‘Arthroplasties, Replacement, Hip’ ab, ti 0

#16 ‘Arthroplasty, Hip Replacement’ ab, ti 3

#17 ‘Hip Prosthesis Implantation’ ab, ti 26

#18 ‘Hip Prosthesis Implantations’ ab, ti 4

#19 ‘Implantation, Hip Prosthesis’ ab, ti 0

#20 ‘Implantations, Hip Prosthesis’ ab, ti 1

#21 ‘Prosthesis Implantation, Hip’ ab ti 1

#22 ‘Prosthesis Implantations, Hip’ ab ti 1

#23 ‘Hip Replacement Arthroplasty’ ab ti 131

#24 ‘Replacement Arthroplasties, Hip’ ab ti 0

#25 ‘Replacement Arthroplasty, Hip’ ab ti 1

#26 ‘Arthroplasties, Hip Replacement’ ab ti 0

#27 ‘Hip Replacement Arthroplasties’ ab ti 16

#28 ‘Hip Replacement, Total’ ab ti 45

#29 ‘Replacement, Total Hip’ ab ti 39

#30 ‘Hip Replacements, Total’ ab ti 4

#31 ‘Replacements, Total Hip’ ab ti 2

#32 ‘Total Hip Replacements’ ab ti 1881

#33 ‘Total Hip Replacement’ ab ti 9923

#34 ‘Total Hip arthroplasty’ ab ti 19065

#35 #14 OR #15 OR #16 OR #17 OR #18 OR #19 OR #20 OR #21 OR #22 OR #23 OR #24 OR #25 OR #26 OR #27 OR #28 OR #29 OR #30 OR #31 OR #32 OR #33 OR #34 28952

#36 #6 AND #13 AND #35 327
