# Supplementary material for: Comparing total hip arthroplasty and hemiarthroplasty for the treatment of displaced femoral neck fracture in the active elderly over 75 years old: a systematic review and meta-analysis of randomized control trials
Source: J Orthop Surg Res. 2020 Jun 11;15:215. doi: 10.1186/s13018-020-01725-3 (PMC7291510; doi:10.1186/s13018-020-01725-3)
Supplement: Supplementary file 2 — Additional file 2. Summary of the quality assessments of key outcomes based on the GRADE approach. We summarised the key comparisons in tables according to the Grading of Recommendations Assessment (GRADE). [file 13018_2020_1725_MOESM2_ESM.docx]

**Additional files 2**

We summarised the key comparisons according to Grading of Recommendations Assessment (GRADE): HHS pain within one year, HHS pain from one to five years, HHS total within one year, HHS total from one to five years, general complications, dislocation within six months, dislocation within five years and dislocation more than five years.


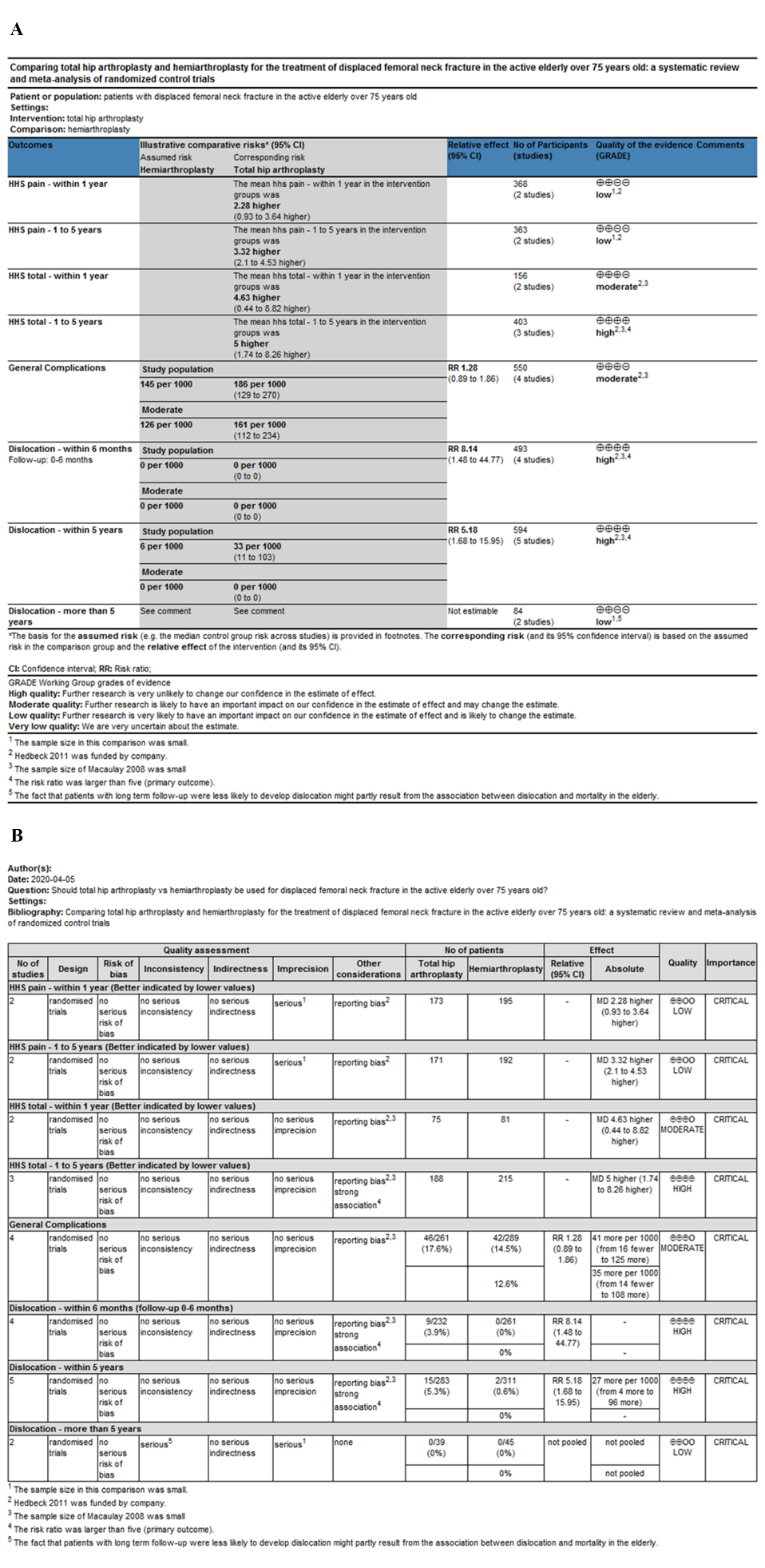


Fig. A Summary of the quality assessments of key outcomes based on the GRADE approach.
